# Comedications Associated with Immune‐Related Adverse Events from Immune‐Checkpoint Inhibitors

**DOI:** 10.1002/cpt.3721

**Published:** 2025-06-16

**Authors:** Léonard Laurent, Baptiste Abbar, Kevin Bihan, Elise Dumas, Floriane Jochum, Bénédicte Lebrun‐Vignes, Jean‐Philippe Spano, Joe‐Elie Salem, Anne‐Sophie Hamy, Fabien Reyal, Paul Gougis

**Affiliations:** ^1^ Residual Tumor & Response to Treatment Laboratory, RT2Lab, INSERM, U932 Immunity and Cancer Institut Curie, Université Paris Cité Paris France; ^2^ Department of Medical Oncology Pitié‐Salpêtrière Hospital, Sorbonne Université, Assistance Publique ‐ Hôpitaux de Paris (AP‐HP) Paris France; ^3^ Centre d'Immunologie et des Maladies Infectieuses (CIMI‐Paris), Inserm U1135 Sorbonne Université Paris France; ^4^ Department of PharmacologyInstitut National de la Santé et de la Recherche Médicale (INSERM), Assistance Publique ‐ Hôpitaux de Paris (AP‐HP), Clinical Investigation Center (CIC‐1901), Regional Pharmacovigilance Centre Pitié‐Salpêtrière Hospital, Sorbonne Université Paris France; ^5^ Department of Mathematics École Polytechnique Fédérale de Lausanne Lausanne Switzerland; ^6^ Department of Gynecology Strasbourg University Hospital Strasbourg France; ^7^ Department of Medical Oncology Institut Curie, Université Paris Cité Paris France; ^8^ Department of Breast, Gynecological and Reconstructive Surgery Institut Curie, Université Paris Cité Paris France; ^9^ Department of Surgical Oncology Institut Godinot Reims France

## Abstract

Immune‐checkpoint inhibitors (ICI) have revolutionized cancer treatment but are responsible for various immune‐related adverse events (irAE). The impact of non‐anticancer medications (comedications) on irAE occurrence remains largely unexplored. The objective of this study was to assess comedications associated with an increased reporting of irAE with ICIs. In this pharmacovigilance study, all individual case safety reports (ICSRs) involving ICIs reported in the World Health Organization international pharmacovigilance database Vigibase up to January 2024 were extracted. All suspect or interacting comedications were analyzed individually and as drug classes using Anatomical Therapeutic Chemical classification level 4. The primary outcome was the reporting odds ratio (ROR) of irAE in patients who received both an ICI and the comedication of interest, compared with ICI‐treated patients who did not receive that comedication. Among 169,753 ICSRs involving an ICI, a total of 314,366 comedications were recorded, with 8,122 identified as “suspect or interacting.” Analysis shows an increased reporting of nephritis with proton pump inhibitors (PPI) (ROR = 29.62 [95% CI = 18.61–47.14]) and with non‐steroidal anti‐inflammatory drugs (ROR = 10.47 [95% CI = 4.15–26.41]), myositis with statins (ROR = 9.41 [95% CI = 3.50–25.30]), ketoconazole with hepatitis (ROR = 20.49 [95% CI = 1.53–274.17]) and autoimmune bullous disease with dipeptyl‐peptidase‐4 inhibitors (ROR = 46.42 [95% CI = 11.71–184.05]), among others. Various drugs, including PPI (ROR = 8.61 [95% CI = 3.48–21.26]), some anti‐infectives (sulfamethoxazole, ROR = 31.31 [95% CI = 13.32–73.61], clavulanic acid, ROR = 18.12 [95% CI = 4.77–68.89]), allopurinol (ROR = 57.11 [95% CI = 11.27–289.39]) or levetiracetam (ROR = 14.91 [95% CI = 2.15–103.64]) were associated with serious cutaneous adverse reactions. Complementary analysis showed higher ROR in the ICI population versus without ICI for the association of nephritis with ibuprofen (ROR_ICI_ = 27.82 *vs.* ROR_VigiBaseWithoutICI_ = 3.56, ROR_ratio_ = 7.81 [95% CI = 1.23–49.50]) and myocarditis with influenza vaccine (ROR_ICI_ = 22.74 vs. ROR_VigiBaseWithoutICI_ = 0.66, ROR_ratio_ = 34.45 [95% CI = 1.66–723.24]), suggesting a synergistic toxicity. This study identified multiple comedications associated with an increased reporting of specific irAE. Some of them might be synergistic warranting further investigation.

**Study Highlights**

**WHAT IS THE CURRENT KNOWLEDGE ON THE TOPIC?**

Immune‐checkpoint inhibitors (ICIs) have revolutionized cancer treatment but are associated with immune‐related adverse events (irAEs). Cancer patients often require multiple non‐antitumor comedications due to underlying comorbidities. While certain anticancer therapies combined with ICIs are linked to specific irAEs, the role of non‐antitumor comedications in modulating irAE occurrence remains unclear.

**WHAT QUESTION DID THIS STUDY ADDRESS?**

This pharmacovigilance study investigated whether the use of comedications alongside ICIs is associated with specific irAEs in cancer patients.

**WHAT DOES THIS STUDY ADD TO OUR KNOWLEDGE?**

This study is the first to investigate the association between any comedication and any irAE using a large‐scale database. It found that multiple irAEs are significantly associated with certain specific comedications. Of note, the association of nephritis with NSAIDs and myocarditis with the influenza vaccine was significantly enriched with ICI compared to other non‐ICI ICSRs, suggesting a synergistic toxicity. A broad range of medications was numerically enriched for serious cutaneous adverse reactions (SCAR), although the significance of these associations warrants further investigation. The signals identified in this research should be confirmed and mechanistically elucidated to better understand how comedications could trigger specific irAEs.

**HOW MIGHT THIS CHANGE CLINICAL PHARMACOLOGY OR TRANSLATIONAL SCIENCE?**

Although clinical pharmacologists are fully aware of the risk of cytochrome‐mediated drug–drug interactions, these results highlight that pharmacodynamic drug–drug interactions could be of importance in assessing the increased frequency of irAE emergence from associated comedications when prescribed with ICIs. Incorporating this knowledge with cautious prescription could enhance patients' safety and guide future medication reconciliation.

Immune‐checkpoint inhibitors (ICI) have revolutionized cancer treatment and are now widely used to treat various malignancies.^1^ However, ICI can also disrupt immune tolerance, leading to immune‐related adverse events (irAE).^2^ Cancer patients often have additional comorbidities, resulting in the concurrent use of antitumor treatments and various non‐anticancer medications (comedications). Understanding the interactions between ICI and other drugs is crucial for ensuring patient safety and optimizing therapeutic outcomes.^3^

Previous studies suggested that other anticancer treatments, when used with ICI, could trigger specific irAE.^4^, ^5^ Certain antitumor treatments are, therefore, not recommended in combination with ICI or following their administration. However, data on the potential risks of increased reporting of irAE from the concomitant use of ICI with non‐antitumor medications remain poorly explored and present contradictory results.^6^, ^7^, ^8^

Since 1967, spontaneous individual case safety reports (ICSRs) from post‐marketing use have been submitted to VigiBase, the WHO's global repository of individual case safety reports that now encompasses over 30 million reports as of January 2024 from over 130 countries. In 2023, we used VigiBase to conduct a comprehensive worldwide pharmacovigilance study, detailing the spectrum, characteristics, and evolution of irAE reporting in the last decade.^2^ This study identified multiple factors influencing the reporting frequency and clinical nuances for 25 subtypes of irAE, providing signals for clinical practice and future research.^2^

In this study, we used the updated database to conduct a disproportionality analysis, investigating the potential association of specific irAEs linked to suspect comedications in patients receiving ICIs.

## MATERIALS AND METHODS

### Data source

This cohort study used all VigiBase ICSRs associated with an ICI classified as “suspect” or “interacting” from 2008 (date of 1st ICI report) to January 1, 2024. ICSRs mentioning ICI not FDA‐approved as of January 2024 (**Figure** **S1**) were excluded. The use of concurrent anticancer therapies, including antiangiogenic agents or cytotoxic drugs, was not an exclusion criterion. For combination regimens, a report mentioning several ICIs was considered on ICI‐combination, irrespective of the timing of administration, which could have been sequential. The full crude extract includes key variables such as case identifiers, patient demographics, seriousness criteria, and fatality status. It provides details on reported drugs (role, dose, regimen, administration route, and actions taken) and adverse reactions (MedDRA term, onset, outcome). Further details are available in the **Supplementary Material**.

### Definitions of immune‐related adverse events

We used Medical Dictionary for Regulatory Activities (MedDRA) (version26.1) to categorize irAE into the 25 entities with narrow definition, as previously described (**Table** **S1**).^2^ These 25 distinct entities are anemia, arthritis, cholangitis, diabetes, encephalomyelitis, enterocolitis, esogastritis, hepatitis, hypophysitis, meningitis, myasthenia gravis‐like syndrome (thereafter myasthenia gravis), myocarditis, myositis, nephritis, pancreatitis, peripheral neuropathy, pneumonitis, sarcoidosis, severe cutaneous adverse reactions (SCAR), skin bullous autoimmune reactions, thrombopenia, thyroiditis, uveitis, vitiligo, and various skin reactions (other than vitiligo, SCAR and skin bullous reaction). Additionally, we created a miscellaneous category, grouping all other rarer irAEs under “other irAEs” (**Table** **S1**) for the list of preferred terms used to determine each narrow irAE, focusing more specifically on an auto‐immunity related term.

### Identification of comedications

ICSR submitters can label a drug as “suspect” or “interacting.” We classified non‐ICI drugs using the WHO ATC system, excluding anticancer drugs (ATC L01, L02, L03) to focus on non‐antitumor comedications. Molecules labeled as “topical” were excluded based on administration route. We analyzed only comedications identified as “suspect” or “interacting” for irAE association. Analyses were conducted at ATC level 5 (individual molecule) or grouped at ATC level 4.

### Statistical analysis

#### Selection of irAE‐comedication tandems

We performed a disproportionality analysis to evaluate the association between the 25 irAE reporting and exposure to comedication and comedication classes. We determined the reporting odds ratio (ROR), defined as the ratio of the odds of the studied irAE with ICI and the studied comedication to the odds of this irAE with ICI without the studied comedication (cf. **Figure** **S3** **&** **Supplementary Materials**).^9^ Each comedication was paired with each irAE thereby constituting the irAE‐comedication‐tandem of interest with its corresponding ROR. All confident intervals (CI) were adjusted for multiple testing using the number of assessed tandems using a Bonferroni method. We estimated the “Percentage used as irAE treatment” as the proportion of ICSRs where the drug's indication matched an irAE symptom (cf. **Supplementary Materials**). To refine our analysis, we applied several exclusion criteria to over‐reported irAE‐comedication tandems. (i) We excluded candidate tandems where less than 50% of ICSRs identified the comedication as the primary suspect drug for the irAE. For instance, in hepatitis cases, rifampicin was reported as a suspect drug alongside isoniazid in 88.9% of ICSRs. The ROR for hepatitis with rifampicin was 9.71, lower than the ROR of 12.63 for isoniazid. Since the rifampicin‐hepatitis tandem was rarely the primary suspect and rifampicin within hepatitis ICSRs often associated with isoniazid, we excluded the rifampicin‐hepatitis tandem from further analysis for hepatitis to avoid confounding effects. (ii) We discarded tandems where the comedication was used to treat the irAE (e.g., pyridostigmine for myasthenia) if the percentage for irAE treatment exceeded 1%. (iii) Comedication classes with identical ICSR counts at over‐reported ATC level 4 and level 5 (signal redundancy) were retained only at level 5.

#### Complementary analyses

To explore a possible synergistic effect between comedications and ICIs in the development of irAEs, we calculated the ROR for each significantly over‐reported irAE‐comedication tandem based on our disproportionality analysis conducted across the entire VigiBase. This analysis excluded ICI‐associated cases and utilized the same MedDRA term to define the irAE (see **Table** **S1**). This analysis provided ROR_VigiBaseWithoutICI_ for a population exposed to the same comedications but not to ICIs. We then calculated the ratio of these RORs (ROR_ratio_ = ROR_ICI_/ROR_VigiBaseWithoutICI_), along with its confidence interval. A synergistic interaction was considered likely when the lower bound of the 95% CI of the ROR_ratio_ (ROR_ratio_^025^) was >1. The standard error (SE) of the ROR_ratio_ was calculated by combining the SEs of the two RORs. The combined SE was used to determine the confidence interval of the ROR_ratio_. A Bonferroni adjustment was applied. Furthermore, we conducted a stepwise multivariate logistic analysis to minimize the Bayesian Information Criterion for final variable selection, applying the Haldane‐Anscombe correction as needed.

Statistical analyses were performed using R version 4.3.2, following STROBE criteria for observational cohorts and READUS‐PV guidelines for disproportionality analyses.^10^

## RESULTS

### Report characteristics

A total of 169,753 ICSRs were extracted from VigiBase, with 314,366 comedications. Of these, 40,589 ICSRs identified at least one comedication, and 4,521 ICSRs (8,122 comedications) were classified as “suspect or interacting.” The flow‐chart of comedication‐irAE tandems is shown in **Figure** **S2**. ICSR characteristics are summarized in **Table** **1**, with 60.2% male (2,615/4,342) and a mean age of 64.8 ± 12.5 years. The majority of cases were from Europe (1,714/4,521; 37.9%), North America (1,805/4,521; 39.9%), and East Asia (789/4,521; 17.5%). Patients received anti‐PD1 or anti‐PD‐L1 monotherapy in 68.6% (3,101/4,521) and 14.4% (649/4,521) of cases, respectively, while 11.4% (516/4,521) received a combination of anti‐CTLA4 and anti‐PD(L)1 therapies. Anti‐LAG3‐based combinations were rare (5/4,521, 0.1%). The distribution of cancer types and the occurrence of 25 irAE are detailed in **Figures** **S3** and **S4**. The ATC class distribution is described in **Figure** **S5**, with anti‐infectives (ATC class J) showing significant enrichment in the suspect/interacting cohort (20.5% vs. 5.1%, *P* < 10–16).

**Table 1:** Individual case safety reports characteristics

Variable name	Level	Overall, *n* (%)	Reports with any comedication, *n* (%)	Reports with a suspect or interacting comedication, *n* (%)
	Total count	*n* = 169,753	*n* = 40,589	*n* = 4,521
Sex	Female	60,078 (39.2)	15,208 (38.2)	1,727 (39.8)
	Male	93,046 (60.8)	24,581 (61.8)	2,615 (60.2)
	Data available, *n* (%)	153,124 (90.2)	39,789 (98.0)	4,342 (96.0)
Age (years)	Mean (SD)	63.9 (12.8)	65.0 (12.1)	64.8 (12.5)
	Data available, *n* (%)	114,639 (67.5)	33,245 (81.9)	3,671 (81.2)
Country group	Eastern Asia	24,030 (14.2)	5,955 (14.7)	789 (17.5)
	Europe	62,501 (36.8)	16,852 (41.5)	1,714 (37.9)
	North America	62,761 (37.0)	14,955 (36.8)	1,805 (39.9)
	Other Country	20,461 (12.1)	2,827 (7.0)	213 (4.7)
Notifier type	Consumer or Non‐Health Professional	32,251 (19.4)	5,343 (13.4)	581 (13.0)
	Other Health Professional	39,192 (23.6)	9,695 (24.2)	1,059 (23.8)
	Physician or Pharmacist	94,546 (57.0)	24,978 (62.4)	2,817 (63.2)
	Data available, *n* (%)	165,989 (97.8)	40,016 (98.6)	4,457 (98.6)
Reporting year	2016 or before	15,548 (9.2)	4,203 (10.3)	437 (9.7)
	2017–2018	37,831 (22.3)	9,883 (24.3)	1,081 (23.9)
	2018–2019	27,480 (16.2)	6,624 (16.3)	735 (16.3)
	2020–2022	60,647 (35.7)	14,176 (34.9)	1,750 (38.7)
	2023–2024	28,247 (16.6)	5,703 (14.1)	518 (11.5)
Cancer type	Breast & Gynecological	9,910 (6.9)	2,507 (6.6)	308 (7.8)
	Other Cancer	1,081 (0.8)	315 (0.8)	48 (1.2)
	Digestive	12,224 (8.6)	3,525 (9.3)	283 (7.2)
	Endocrine	501 (0.4)	102 (0.3)	20 (0.5)
	Genitourinary	23,446 (16.4)	6,593 (17.4)	607 (15.4)
	Hematological	3,306 (2.3)	983 (2.6)	108 (2.7)
	Nervous	975 (0.7)	349 (0.9)	37 (0.9)
	Head and Neck & Eye	5,177 (3.6)	1,091 (2.9)	133 (3.4)
	Skin	35,638 (25.0)	7,981 (21.1)	937 (23.7)
	Thorax	50,408 (35.3)	14,352 (38.0)	1,467 (37.2)
	Data available, *n* (%)	142,666 (84.0)	37,798 (93.1)	3,948 (87.3)
ICI Regimen	Anti‐CTLA4 alone	11,374 (6.7)	2,056 (5.1)	246 (5.4)
	Anti‐LAG3 combination	366 (0.2)	150 (0.4)	5 (0.1)
	Anti‐PD(L)1 + Anti‐CTLA4	20,379 (12.0)	5,917 (14.6)	516 (11.4)
	Anti‐PD1 (PD1) Alone	109,689 (64.6)	24,167 (59.5)	3,101 (68.6)
	Anti‐PD1 + Anti‐PDL1	180 (0.1)	51 (0.1)	4 (0.1)
	Anti‐PDL1 (PDL1) alone	27,765 (16.4)	8,248 (20.3)	649 (14.4)
Fatal outcome		31,764 (18.7)	8,282 (20.4)	774 (17.0)

Continuous variables were expressed as mean ± SD or median (IQR), and categorical variables as number (percentage). CTLA4, cytotoxic T‐lymphocyte‐associated protein 4; ICI, Immune Checkpoint Inhibitors; LAG3, lymphocyte activation gene 3; PD1, programmed cell‐death 1; PDL1, programmed cell‐death ligand.

### IrAEs and comedications

**Figure 1:**
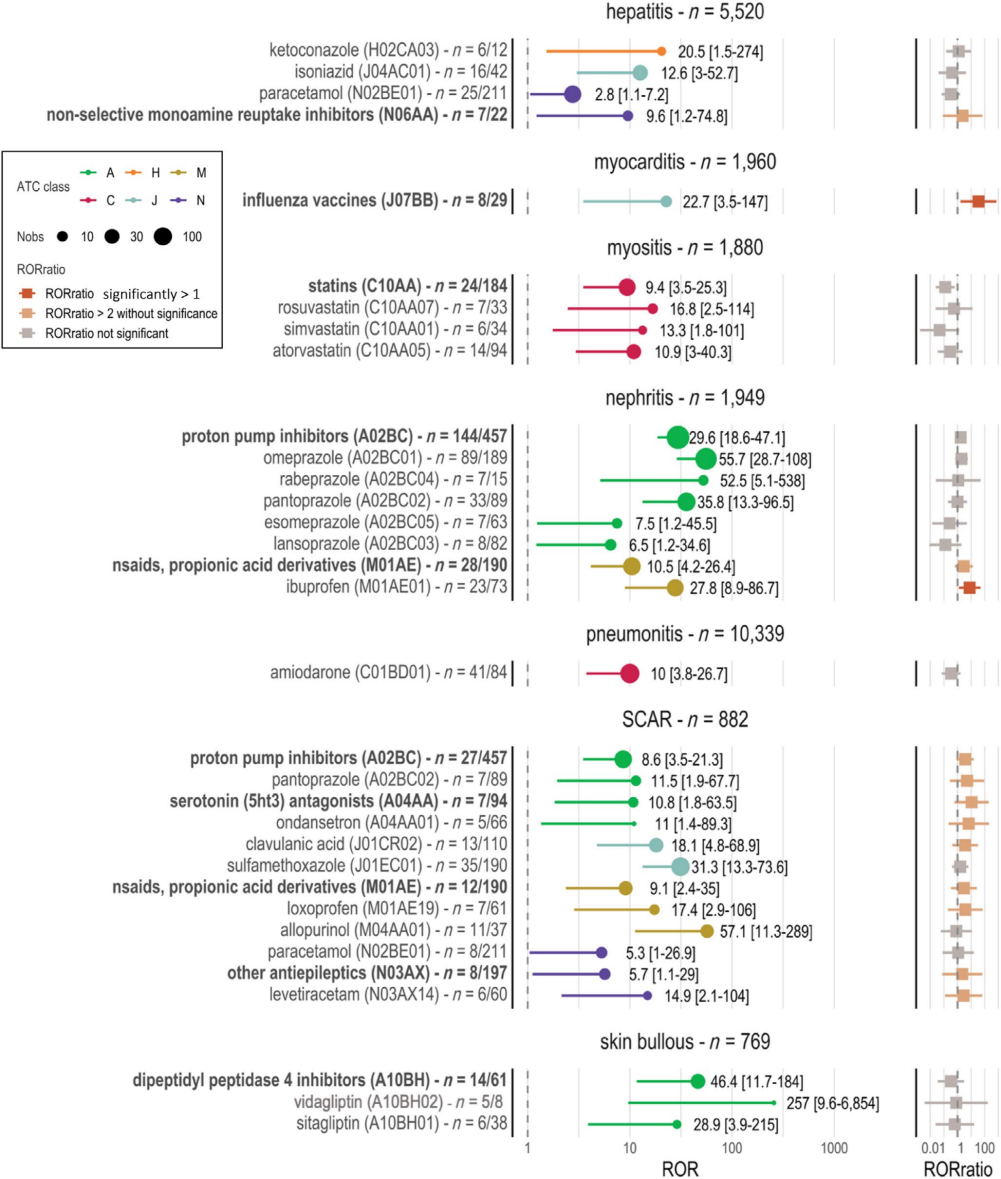
Reporting Odds ratios for ATC level 4 & 5 for “suspect or interacting” comedications and corresponding ROR_ratio_. The figure displays the reporting odds ratios (ROR, left) for ATC (Anatomical Therapeutic Chemical) classification level 4 and 5 comedications, significantly associated with irAEs. Only irAE‐comedication tandems with statistically significant associations are represented. Excluded comedications: used as irAE occurrence treatment, mainly co‐reported with other over‐reported drugs, and ATC level 4 with the same signal as ATC level 5 (**Figure** **S1**). ROR_ratio_ (right) is computed using the ratio of the ROR of comedication and irAE within the ICI database (ROR represented on the left) and the ROR of the same adverse events within all VigiBase excluding reported with ICI. Each value used for the calculation of the ROR_ratio_ is available in **Table** **S3**.

Multiple tandems of comedications with increased irAE reporting (ROR > 1) were identified for various irAE. Nephritis was strongly associated with the proton pump inhibitors (PPI) (ROR = 29.62 [95% CI = 18.61–47.14]) and propionic acid non‐steroidal anti‐inflammatory drugs (NSAID) (ROR = 10.47 [95% CI = 4.15–26.41]). Myositis was associated with statins (ROR = 9.41 [95% CI = 3.50–25.30]) and particularly with rosuvastatin (ROR = 16.76 [95% CI = 2.47–113.79]). Hepatitis was associated with systemic ketoconazole (ROR = 20.49 [95% CI = 1.53–274.17]) and isoniazid (ROR = 12.63 [95% CI = 3.03–52.69]). Myocarditis was associated with influenza vaccines (ROR = 22.74 [95% CI = 3.51–147.37]). Pneumonitis was associated with amiodarone (ROR = 10.01 [95% CI 3.75–26.71]). SCARs were linked with numerous drugs, including allopurinol (ROR = 57.11 [95% CI = 11.27–289.39]), sulfamethoxazole (ROR = 31.31 [95% CI = 13.32–73.61]). Finally, dipeptidyl peptidase‐4 inhibitors (DPP4i) were associated with autoimmune bullous diseases (ROR = 46.42 [95% CI = 11.71–184.05]), with vildagliptin showing an extreme association (ROR = 256.79 [95% CI = 9.62–6,853]). Detailed results are presented in **Figure** **1** and **Table** **S3**.

### Sensitivity and multivariable analysis

The synergy test using the ROR_ratio_ was significant for two tandems: influenza vaccines (ATC level 4) with myocarditis (ROR_ratio_ = 34.45 [95% CI = 1.66–723.24]) and ibuprofen with nephritis (ROR_ratio_ = 7.81 [95% CI = 1.23–49.50]). Detailed results for each pair are presented in **Figure** **1** and **Table** **S4**. Most comedications associated with SCAR had a ROR_ratio_ of 2 or more, although the ROR_ratio_ did not reach statistical significance.

We performed a multivariate logistic regression on the 33 tandems with suspected interaction. The adjustment factors for this regression were those identified in our previous work,^2^ with the inclusion of the tested comedication. No change was observed in the significance of the tested tandems in the multivariate analysis compared to the univariate analysis (**Table** **S5**).

A sensitivity analysis was performed. We excluded ICSRs where another anticancer drug was co‐reported alongside ICIs, retaining only cases where ICIs were the sole anticancer agents. This reduced the number of comedications analyzed from 8,122 to 5,383 (**Figure** **S6**). Findings were consistent with the main analysis, except for an increased reporting of atorvastatin with hepatitis (ROR = 4.70 [95% CI = 1.05–20.97]), while previously observed signals for selective serotonin reuptake inhibitors with hepatitis and rosuvastatin with myositis were no longer detected. Additionally, we analyzed all drugs labeled as “concomitant” (**Figure** **S7**). Most signals overlapped with the “suspect/interacting” analysis, except for vonoprazan with colitis (ROR = 10.21 [95% CI = 1.93–54.07]) and multiple drugs (several proton pump inhibitors, alpha glucosidase inhibitors, benzodiazepines) with pneumonitis. However, no increased reporting was observed for influenza vaccines with myocarditis, statins with myositis, or NSAIDs with nephritis.

There were no significant fatality rate differences with myositis with concomitant statins (25%, *N* = 6/24) compared to ICSR without statins (21.93%, *N* = 407/1856), (*P* = 0.91).

## DISCUSSION

In this cohort study, we analyzed comedications associated with an increased reporting of specific irAE classified in 25 subtypes. We identified several liable comedications, with some potentially exhibiting a synergistic effect with ICI that would trigger myocarditis and nephritis.

The association of certain comedications with previously known adverse drug reactions (ADRs) in ICI‐treated patients has been scarcely studied, mostly in small retrospective cohorts or case reports, such as statins with myositis and PPI/NSAID with nephritis.^6^, ^7^, ^8^, ^11^ This VigiBase analysis stands out due to its global scope and comprehensive evaluation of all possible irAE/comedication combinations. The significant RORs indicating an increased reporting of irAE‐comedication tandems in our disproportionality analysis align with previously known drug‐ADR associations. Robust class effects were observed for the use of statins and the increased reporting of myositis, and for PPIs and NSAIDs with nephritis. We also identified interactions, such as autoimmune bullous disease with DPP4i, which were previously reported only in isolated case studies.^12^ Statins, PPIs, and DPP4 inhibitors can induce immune‐mediated adverse effects that may be exacerbated by ICIs. Statins are known to cause myopathy through direct muscle toxicity and immune pathways.^13^ A genetic variant in the LILRB5 gene (rs12975366>C) is linked to elevated muscle injury markers (CK, LDH) and increased reporting of statin‐induced myopathy.^14^ This process may result from altered T‐cell (Treg) function, leading to heightened immune responses against muscle tissue. Some patients develop autoantibodies against HMG‐CoA reductase, causing immune‐mediated necrotizing myopathy unresponsive to statins but responsive to immunosuppressive therapy.^13^ Similarly, PPIs may induce acute interstitial nephritis through immune‐mediated mechanisms by acting as haptens, binding to the tubular basement membrane and triggering immune responses.^15^ Since ICIs can independently cause nephritis, they might trigger this effect. Finally, DPP4i antidiabetic agents have been linked to an increased reporting of bullous pemphigoid, potentially by altering keratinocyte gene expression and upregulating interleukin‐6, promoting inflammatory cascades related to bullous pemphigoid severity.^16^

Furthermore, to evaluate potential synergistic effects of comedications with ICIs, we performed an additional analysis using the ROR_ratio_. The ROR_ratio_ compares the occurrence of irAE‐comedication pairs in ICI‐exposed patients to non‐exposed ones, with the aim of identifying the strongest signals of potential synergy. The ROR_ratio_ analysis suggests two tandems with significant synergy: nephritis with ibuprofen and myocarditis with the influenza vaccine in ICI‐treated patients. Previous studies indicate an increased risk of nephritis with NSAIDs and ICI,^11^ and myocarditis with influenza vaccinations.^17^, ^18^ Vaccinations can stimulate the immune system, leading to transient elevations in inflammatory cytokines and, in some rare instances, to myocarditis.^18^ This cytokine surge, when combined with ICIs, may enhance the occurrence of myocarditis. These results reinforce the caution that should be exerted toward NSAIDs. Further research is required to validate the potential myocarditis risk in ICI‐treated patients receiving the influenza vaccine. Several medications, including PPIs, 5HT3 antagonists, NSAIDs, clavulanic acid, and levetiracetam, showed a ROR_ratio_ exceeding 2 for SCARs, despite individual significance. Normally, PD‐L1 expression on keratinocytes protects against inflammatory damage, but ICIs may increase vulnerability by disrupting the PD‐1/PD‐L1 pathway, favoring autoreactive CD8+ T‐cells targeting keratinocytes, and eventually leading to severe cutaneous reactions.^19^ The broad range and substantial number of medications associated with elevated ROR_ratio_ should lead to further investigations. Findings from our pharmacovigilance study do not warrant direct changes in prescribing, as some treatments remain protective. However, they should be incorporated into the broader evidence base guiding patient‐centered precision medicine, particularly regarding two tandem pairs with a potential synergy signal.

This study has limitations inherent to pharmacovigilance data collection. Small sample sizes for certain tandems, result in wide 95% confidence intervals calling for cautious interpretation of the observed disproportionality. Despite multiple adjustments, certain biases remain: underreporting of suspected adverse drug reactions and concomitant medications is a known limitation but is likely less pronounced for serious adverse events.^20^ Fluctuating ADR reporting in a drug's early post‐marketing period may influence our signal, though its precise impact is difficult to quantify.^21^ Although multivariate analysis controlled for baseline differences, ROR findings should be interpreted cautiously, as patients with and without the comedications of interest may still differ significantly. Certain associations, like statins with myositis, PPIs/NSAIDs with nephritis, and DPP4 inhibitors with bullous skin disorders, also occur outside the ICI setting.^15^, ^16^, ^22^ We minimized biases in identifying comedications requiring heightened vigilance by testing tandem synergies, prioritizing “suspect or interacting” comedications, and applying multiple testing corrections to our statistical analyses. Focusing on suspect or interacting molecules enhances signal reliability and reduces biases, yielding reliable findings but limiting signal discovery. However, ROR_ratio_ analysis has not been validated outside of the present research and remains a highly exploratory approach.

PPIs and NSAIDs, both linked to increased reporting of nephritis in the ICI setting, warrant distinct prescription considerations. For PPIs, which are frequently overused, these findings underscore the need for more risk‐aware prescribing. For NSAIDs, already restricted in cancer patients due to known nephrotoxicity, the observed signals further encourage curtailing them in favor of safer alternatives. As for potential synergistic myocarditis with influenza vaccines, further investigations are needed before contraindicating their use in ICI‐treated patients in clinical practice.

## FUNDING

This study was supported by the Contrats ED: Programme Blanc Institut Curie Paris‐Sciences Lettres University Academic Program (Dr Gougis) and the Fondation ARC Pour la Recherche Sur le Cancer (Dr Abbar). The Science Humaine et Sociale, Institut National du Cancer academic program SHS grant, Sanofi iTech award, and Monoprix supported the Institut Curie Residual Tumor and Response to Treatment Laboratory research group (Drs Laurent, Gougis, Hamy, Jochum, and Prof. Reyal). Role of the Funder/Sponsor: The funders had no role in the design and conduct of the study; collection, management, analysis, and interpretation of the data; preparation, review, and decision to submit the manuscript for publication.

## CONFLICT OF INTEREST

Baptiste Abbar reports consulting fees or honoraria from Novartis, AstraZeneca, BMS, MSD, Astellas, and Sanofi. Kevin Bihan reports consulting fees for Medinspire. Joe‐Elie Salem reports consultancy fees or grant support from Novartis, BMS, BeiGene, and Banook Group. Jean‐Philippe Spano reports consultant or advisory role fees from Roche, MSD, BMS, Lilly, AstraZeneca, Daiichi‐Sankyo, Mylan, Novartis, Pfizer, PFO, LeoPharma, and Gilead and research Grant for MSDAvenir. Paul Gougis reports consulting fees for BMS, an academic grant from Sanofi, and travel accommodation by Eisai. All other authors declared no competing interests for this work.

## AUTHOR CONTRIBUTIONS

P.G., F.R., A.‐S.H., J.‐E.S., J.‐P.S., B.L.‐V., F.J.: designed research. P.G., L.L., B.A.: wrote manuscript. P.G., L.L., A.‐S.H., K.B.: performed research. P.G., L.L., E.D.: analyzed data.

## Supporting information


Data S1

## Data Availability

Publicly available datasets were analyzed in this study. The data can be obtained from: www.vigiaccess.org. This study has not been published or presented elsewhere. The information does not represent the opinion of the Uppsala Medical Center or the World Health Organization.
