# Supplementary material for: Comedications Associated with Immune‐Related Adverse Events from Immune‐Checkpoint Inhibitors
Source: Clin Pharmacol Ther. 2025 Jun 16;118(3):593–9. doi: 10.1002/cpt.3721 (PMC12355013; doi:10.1002/cpt.3721)
Supplement: Supplementary file 1 — Data S1 [file CPT-118-593-s001.docx]

**Supplementary Materials**

Comedications associated with immune-related adverse events from immune-checkpoint inhibitors

[Supplementary Materials and Methods 2](#_Toc193738099)

[Statistical Analysis 2](#_Toc193738100)

[Selection of comedication-irAE tandems 2](#_Toc193738101)

[Caveat Uppsala Monitoring Center 2](#_Toc193738102)

[Supplementary Figures 4](#_Toc193738103)

[Figure S1. Flow-chart of extracted ICSRs from VigiBase 4](#_Toc193738104)

[Figure S2. Flow-chart of the comedications within the extracted ICSRs from VigiBase 5](#_Toc193738105)

[Figure S3. Treemap illustrating the distribution of cancers associated with an irAE in VigiBase 6](#_Toc193738106)

[Figure S4. Treemap illustrating the 25 predefined irAEs and their count within the extracted ICSRs 7](#_Toc193738107)

[Figure S5. Distribution of comedications 8](#_Toc193738108)

[Figure S6. Reporting Odds ratios for ATC level 4 & 5 comedications excluding ICSRs with co-reported anticancer drugs 9](#_Toc193738109)

[Figure S7. Reporting Odds ratios for ATC level 4 & 5 for “concomittant” comedications 10](#_Toc193738110)

[Supplementary Tables 11](#_Toc193738111)

[Table S1. MedDRA (Medical dictionary for regulatory activities) preferred terms (PT) used to classify the narrow definitions of immune related adverse events (irAE, 25 specific and one for others) 11](#_Toc193738112)

[Table S2. The Strengthening the Reporting of Observational Studies in Epidemiology (STROBE) checklist: guidelines for reporting observational studies. 14](#_Toc193738113)

[Table S3. Over-reported tandems 15](#_Toc193738114)

[Table S4. Calculation of the reporting odds ratio (ROR) ratio (ROR_ratio_). 17](#_Toc193738115)

[Table S5. Multivariate analysis performed on the relevant associations identified. 18](#_Toc193738116)

# Supplementary Materials and Methods

## Statistical Analysis

### Selection of comedication-irAE tandems

We performed a disproportionality analysis to evaluate the association between irAE occurrence and exposure to comedication. Once comedications (ATC level 4,5) and irAEs were defined, each combination (tandem) was tested to determine its reporting odds ratio (ROR). ROR was defined as the ratio of the odds of the irAE of interest with exposure to ICI and comedication to the odds of each irAE with exposure to ICI without comedication. A comedication was considered over-reported with an irAE when the lower bound of the ROR CI95% (ROR_025_) was over 1. Adjustment on the number of tandem tested was performed using Bonferroni’s correction. Occurrence of 4 or less tandems was considered non-significant.

Comedications that were co-reported with another suspect comedication were considered “main” when they had the highest ROR value of all comedications for the irAE.

We estimated the the “Percentage used as irAE treatment” by the percentage of ICSRs (reports) where the drug indication was a symptom of an irAE (defined as “broad irAE in [PMID:38560659]).

Over-reported tandems were then excluded when:

- Tandems for which the percentage of ICSRs was used as main was <50% were discarded. Rifampicin was mainly (88.9%) reported with isoniazid for hepatitis and its ROR for hepatitis was 9.71 (<12.63 for isoniazid).
- Tandems with a comedications used as a treatment of the irAE (such as pyridostigmine for myasthenia) were discarded (percentage cut-off = 1%).
- Comedications with the exact same N with ICI of ATC level 4 and ATC level 5, were analyzed only for level 5.

### Caveat Uppsala Monitoring Center

VigiBase is the World Health Organization(WHO) global database of reported potential side effects of medicinal products, developed and maintained by Uppsala Monitoring Centre (UMC).

The information presented in this research and manuscript does not represent the opinion of the UMC or the WHO.

**Overview of VigiBase Data, collected variables and Classification Criteria for ICSRs**

The full crude extract gathers notably:

- A list of the unique **cases**, containing general administrative information (unique identifier of the report, country of origin, date of reporting, and reporter qualification), patient characteristics (gender, age, weight, height), the seriousness criteria of the report and its fatality, study names when appliable, and total number of Suspect/Interacting drugs.
- A detailed list of the **drugs** involved in the report, its role (concomitant / suspect / interacting), its indication, the dose, the dosage regimen, the route of administration, the start and end date, the action taken with drug.
- A list of the **reactions**, with the term reported to Upsala monitoring center and the MedDRA preferred term, a start and end date, and the outcome of the reaction (Died/Recovered with or without sequelae/Not recovered)
- A list of **drug – reaction link** with the time-to-onset, information on a rechallenge performance / Reaction resolution, information on a rechallenge performance / recurrence of the reaction, and a causality assessment with method and source.

A report mentioning multiple ICIs was classified as an ICI-combination regimen, regardless of whether the ICIs were administered concurrently or sequentially.

Fatal outcomes and seriousness are reported as independent variables within VigiBase. All ICSRs having “Death” as the cause of serious adverse events were reported as “Fatal”.

# Supplementary Figures

## Figure S1. Flow-chart of extracted ICSRs from VigiBase

ICSRs were extracted from VigiBase based on the presence of a defined irAE (Figure S4) and an Immune Checkpoint Inhibitor.

The reported Immune Checkpoint Inhibitors are atezolizumab (anti-PDL1), avelumab (anti-PDL1), cemiplimab (anti-PD1), dostarlimab (anti-PD1), durvalumab (anti-PDL1), ipilimumab (anti-CTLA4), nivolumab (anti-PD1), pembrolizumab (anti-PD1), REGN 2810 (anti-PD1), relatlimab (anti-LAG3), retifanlimab (anti-PD1), tislelizumab (anti-PD1), toripalimab (anti-PD1), and tremelimumab (anti-CTLA4).


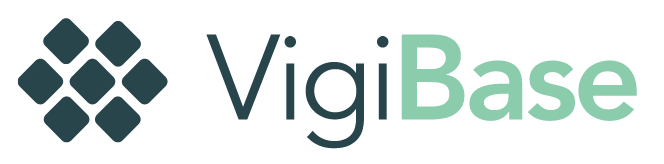


Abbreviations: ICI: Immune Checkpoint Inhibitors; FDA: Food and Drug Administration.

## Figure S2. Flow-chart of the comedications within the extracted ICSRs from VigiBase

Comedications reported as "suspect or interacting" were selected at ATC levels 4 and 5 from the extracted ICSRs (Supplemental Figure S1). These comedications were paired with irAEs to form N tandems (comed-irAE), which were then analyzed. Only tandems significantly over reported, not meeting any exclusion criteria, were conserved. To address alpha risk inflation, we used the Bonferroni correction for multiple comparisons based on the number of tandems tested. In a sensitivity analysis excluding ICSRs with co-reported anticancer drugs (Figure S6), the number of comedications analyzed decreased from 8,122 comeds to 5,383 comeds.

*Abbreviations: ATC: Anatomical Therapeutical Chemical classification; comed: comedication; ROR025 : Reporting Odds Ratio lower bound of the 95% confidence interval (CI)*

## Figure S3. Treemap illustrating the distribution of cancers associated with an irAE in VigiBase

Overall number of irAE per organ system.

Abbreviations: NSCLC : Non-Small Cell Lung Cancer; SCLC: Small Cell Lung Cancer; lung NOS: lung Not Otherwise Specified.

## Figure S4. Treemap illustrating the 25 predefined irAEs and their count within the extracted ICSRs

Twenty-five irAEs were predefined (Figure S5), and their distribution within the extracted ICSRs is presented, categorized by organ system.

## Figure S5. Distribution of comedications

*A. & B. Description of the medications without anticancer purposes (comedications) present in all* ICSRs *(reported as concomitant or suspect or interacting, “all comedications”, panel A, n= 314,366) and comedications reported as “suspect or interacting” included in the analysis (“suspect or interacting”, panel B, n=8,122). Each molecule class (WHO Anatomical Therapeutic Chemical classification level 4) was grouped by ATC level 1. Size of cells is proportional to the count.
C. Proportion of comedications for each ATC level 1 within all comedications (left) versus "suspected or interacting" comedications (right).*

*Abbreviations: all: all comedications; NSAID: non-steroidal anti-inflammatory drugs SI: Suspect or Interacting; WHO: world health organization.*

## Figure S6. Reporting Odds ratios for ATC level 4 & 5 comedications excluding ICSRs with co-reported anticancer drugs

Comedications reported as "suspected or interacting" were selected at ATC levels 4 and 5 from the extracted ICSRs (Supplemental Figure S1). ICSRs mentioning a co-reported anticancer drugs were excluded at this point, resulting in 5,383 comedications. Comedications were paired with irAEs to form N tandems (comed-irAE), which were then analyzed. Only tandems significantly over reported, not meeting any exclusion criteria, were conserved. To address alpha risk inflation, we used the Bonferroni correction for multiple comparisons based on the number of tandems tested.

## Figure S7. Reporting Odds ratios for ATC level 4 & 5 for “concomittant” comedications

Comedications reported as "concomittant" were selected at ATC levels 4 and 5 from the extracted ICSRs (Supplemental Figure S1). Comedications were paired with irAEs to form N tandems (comed-irAE), which were then analyzed. Only tandems significantly over reported, not meeting any exclusion criteria, were conserved. To address alpha risk inflation, we used the Bonferroni correction for multiple comparisons based on the number of tandems tested.

# Supplementary Tables

## Table S1. MedDRA (Medical dictionary for regulatory activities) preferred terms (PT) used to classify the narrow definitions of immune related adverse events (irAE, 25 specific and one for others)

Abbreviations: irAE: immune-related adverse events, PT: Preferred Terms

| **irAE system** | **irAE type** | **MedDRA preferred term (PT)** |
| --- | --- | --- |
| hematology | anemia | Aplastic anaemia, Autoimmune anaemia, Autoimmune aplastic anaemia, Autoimmune haemolytic anaemia, Autoimmune pancytopenia, Haemolytic anaemia, Cold type haemolytic anaemia, Coombs positive haemolytic anaemia, Warm autoimmune haemolytic anaemia |
| rheumatology | arthritis | Ankylosing spondylitis, Arthritis, Arthritis reactive, Arthrotoxicity, Autoimmune arthritis, Dactylitis, Immune-mediated arthritis, Juvenile idiopathic arthritis, Lupus-like syndrome, Nodal osteoarthritis, Oligoarthritis, Polyarthritis, Polymyalgia rheumatica, Psoriatic arthropathy, Rheumatic disorder, Rheumatoid arthritis, Sacroiliitis, Seronegative arthritis, SLE arthritis, Spondylitis, Still's disease, Synovitis, Systemic lupus erythematosus |
| pancreato-hepatic | cholangitis | Autoimmune cholangitis, Biliary cirrhosis, Cholangiolitis, Cholangitis, Cholangitis acute, Cholangitis sclerosing, Immune-mediated cholangitis, Immune-mediated cholestasis, Primary biliary cholangitis, Vanishing bile duct syndrome |
| endocrine | diabetes | Diabetic coma, Diabetic hyperglycaemic coma, Diabetic hyperosmolar coma, Diabetic ketoacidosis, Diabetic ketoacidotic hyperglycaemic coma, Diabetic ketosis, Diabetic metabolic decompensation, Fulminant type 1 diabetes mellitus, Insulin autoimmune syndrome, Ketosis-prone diabetes mellitus, Latent autoimmune diabetes in adults, Pancreatogenous diabetes, Type 1 diabetes mellitus |
| nervous | encephalomyelitis | Acute disseminated encephalomyelitis, Acute haemorrhagic leukoencephalitis, Autoimmune encephalopathy, Central nervous system lupus, CNS ventriculitis, Encephalitis, Encephalitis allergic, Encephalitis autoimmune, Encephalitis brain stem, Encephalitis toxic, Encephalomyelitis, Immune effector cell-associated neurotoxicity syndrome, Immune-mediated encephalitis, Immune-mediated encephalopathy, Limbic encephalitis, Multiple sclerosis, Myelitis, Myelitis transverse, Neuromyelitis optica spectrum disorder, Noninfectious myelitis, Noninfective encephalitis, Secondary cerebellar degeneration, Stiff person syndrome, Toxic encephalopathy, Toxic leukoencephalopathy |
| gastro-intestinal | enterocolitis | Acute haemorrhagic ulcerative colitis, Allergic colitis, Appendicitis, Appendicitis perforated, Autoimmune colitis, Autoimmune enteropathy, Coeliac disease, Colitis, Colitis erosive, Colitis ischaemic, Colitis microscopic, Colitis ulcerative, Complicated appendicitis, Crohn's disease, Diversion colitis, Diverticulitis, Diverticulitis intestinal perforated, Duodenal papillitis, Duodenitis, Enteritis, Enterocolitis, Enterocolitis haemorrhagic, Eosinophilic colitis, Epiploic appendagitis, Erosive duodenitis, Gastroenteritis eosinophilic, Gastrointestinal toxicity, Immune- mediated enterocolitis, Inflammatory bowel disease, Ischaemic enteritis, Mesenteric panniculitis, Necrotising colitis, Neutropenic colitis, Proctitis, Proctitis haemorrhagic, Proctitis ulcerative, Pseudomembranous colitis, Rectal ulcer haemorrhage, Terminal ileitis, Ulcerative duodenitis |
| gastro-intestinal | esogastritis | Chronic gastritis, Eosinophilic oesophagitis, Erosive oesophagitis, Gastritis, Gastritis erosive, Gastritis haemorrhagic, Gastroenteritis eosinophilic, Gastrointestinal toxicity, Haemorrhagic erosive gastritis, Immune-mediated gastritis, Immune-mediated oesophagitis, Ischaemic gastritis, Lymphocytic oesophagitis, Mesenteritis, Necrotising gastritis, Necrotising oesophagitis, Noninfectious peritonitis, Oesophagitis, Oesophagitis haemorrhagic, Oesophagitis ulcerative, Ulcerative gastritis |
| pancreato-hepatic | hepatitis | Autoimmune hepatitis, Cholestatic liver injury, Drug-induced liver injury, Granulomatous liver disease, Hepatic infiltration eosinophilic, Hepatitis, Hepatitis acute, Hepatitis cholestatic, Hepatitis fulminant, Hepatitis toxic, Hepatocellular injury, Hepatotoxicity, Immune-mediated hepatic disorder, Immune-mediated hepatitis, Ischaemic hepatitis, Liver injury, Mixed liver injury |
| endocrine | hypophysitis | Hypophysitis, Immune-mediated hypophysitis, Lymphocytic hypophysitis |
| nervous | meningitis | Arachnoiditis, Meningitis, Meningitis aseptic, Meningitis chemical, Meningitis noninfective, Pachymeningitis |
| myology | myasthenia  gravis-like | Diaphragm muscle weakness, Diaphragmatic disorder, Diaphragmatic paralysis, Eyelid ptosis, Immune-mediated myasthenia gravis, Myasthenia gravis, Myasthenia gravis crisis, Myasthenic syndrome, Neuromuscular blockade, Neuromuscular toxicity, Neuromyopathy, Ocular myasthenia, Orbital myositis, Respiratory muscle weakness |
| myology | myocarditis | Autoimmune myocarditis, Cardiotoxicity, Carditis, Eosinophilic myocarditis, Giant cell myocarditis, Hypersensitivity myocarditis, Immune-mediated myocarditis, Myocarditis, Myopericarditis, Toxic cardiomyopathy |
| myology | myositis | Autoimmune myositis, Dermatomyositis, Diaphragm muscle weakness, Idiopathic inflammatory myopathy, Immune-mediated myositis, Inclusion body myositis, Mitochondrial myopathy acquired, Muscle necrosis, Musculoskeletal toxicity, Myofascitis,Myopathy, Myopathy toxic, Myositis, Myositis-like syndrome, Necrotising myositis, Orbital myositis, Polymyositis, Respiratory muscle weakness, Rhabdomyolysis |
| genitourinary | nephritis | Anti-glomerular basement membrane disease, Autoimmune nephritis, C3 glomerulopathy, Glomerulonephritis, Glomerulonephritis acute, Glomerulonephritis membranoproliferative, Glomerulonephritis membranous, Glomerulonephritis minimal lesion, Glomerulonephritis proliferative, Glomerulonephritis rapidly progressive, Glomerulonephropathy, Goodpasture's syndrome, Henoch-Schonlein purpura nephritis, IgA nephropathy, IgM nephropathy, Immune-mediated nephritis, Immune-mediated renal disorder, Lupus nephritis, Mesangioproliferative glomerulonephritis, Nephritic syndrome, Nephritis, Nephritis allergic, Nephropathy toxic, Nephrotic syndrome, Pyelonephritis, Scleroderma renal crisis, Tubulointerstitial nephritis |
| other irAE | other irAE | Acute graft versus host disease, Acute graft versus host disease in skin, Acute graft versus host disease oral, Chronic allograft nephropathy, Chronic graft versus host disease, Chronic graft versus host disease in liver, Chronic graft versus host disease in skin, Complications of transplanted kidney, Complications of transplanted liver, Complications of transplanted lung, Corneal graft rejection, Graft versus host disease, Graft versus host disease in eye, Graft versus host disease in gastrointestinal tract, Graft versus host disease in liver, Graft versus host disease in lung, Graft versus host disease in skin, Heart transplant rejection, Kidney transplant rejection, Liver transplant rejection, Renal transplant failure, Skin graft failure, Transplant dysfunction, Transplant failure, Transplant rejection, Transplantation complication, Acute sinusitis, Addison's disease, Adrenal gland injury, Adrenalitis, Allergic sinusitis, Alveolar osteitis, Angular cheilitis, Anti-neutrophil cytoplasmic antibody positive vasculitis, Antiphospholipid syndrome, Antisynthetase syndrome, Aortitis, Arteritis, Atrophic glossitis, Atrophic vulvovaginitis, Autoimmune disorder, Autoimmune endocrine disorder, Autoimmune inner ear disease, Autoimmune neutropenia, Autoimmune pericarditis, Autoimmune retinopathy, Autoinflammatory disease, Balanoposthitis, Bartholinitis, Behcet's syndrome, Birdshot chorioretinopathy, Blepharitis, Bursitis, Capillary leak syndrome, Cell-mediated cytotoxicity, Central nervous system vasculitis, Cheilitis, Chemical peritonitis, Cholecystitis, Cholecystitis acute, Cholecystitis chronic, Chondritis, Chorioretinitis, Choroiditis, Chronic eosinophilic rhinosinusitis, Chronic sinusitis, Collagen disorder, Collagen-vascular disease, Conjunctivitis, Conjunctivitis allergic, Connective tissue disorder, Connective tissue inflammation, Coronary artery dilatation, Costochondritis, Cryoglobulinaemia, Cryptitis, Cutis laxa, Cystitis, Cystitis interstitial, Cystitis noninfective, Dacryoadenitis acquired, Diffuse vasculitis, Digital pitting scar, Dressler's syndrome, Dry eye, Emphysematous cystitis, Endocarditis, Endocarditis noninfective, Endometritis, Endophthalmitis, Eosinophilic fasciitis, Eosinophilic granulomatosis with polyangiitis, Eosinophilic pleural effusion, Epicondylitis, Epididymitis, Epiglottitis, Episcleritis, Evans syndrome, Exfoliative rash, Eye allergy, Fasciitis, Giant cell arteritis, Giant papillary conjunctivitis, Gingivitis, Glossitis, Granulomatosis with polyangiitis, Granulomatous lymphadenitis, Haemophagocytic lymphohistiocytosis, Haemorrhagic cholecystitis, Henoch-Schonlein purpura, Hidradenitis, Histiocytic necrotising lymphadenitis, Hypersensitivity vasculitis, Immune system disorder, Immune-mediated adrenal insufficiency, Immune-mediated adverse reaction, Immune-mediated cystitis, Immune-mediated cytopenia, Immune-mediated endocrinopathy, Immune-mediated neurological disorder, Immune-mediated scleritis, Immunoglobulin G4 related disease, Injection site rash, Keratitis, Labyrinthitis, Lacrimation disorder, Laryngitis, Laryngopharyngitis, Livedo reticularis, Lymphadenitis, Lymphangitis, Mastitis, Mastoiditis, Mediastinitis, Meibomianitis, Mixed connective tissue disease, Mucocutaneous rash, Mucosal toxicity, Nasopharyngitis, Neurotoxicity, Noninfective chorioretinitis, Noninfective conjunctivitis, Noninfective gingivitis, Noninfective sialoadenitis, Ocular rosacea, Ocular toxicity, Ocular vasculitis, Oculomucocutaneous syndrome, Optic ischaemic neuropathy, Oral toxicity, Orchitis, Orchitis, Orchitis noninfective, Osteitis, Osteitis deformans, Osteomyelitis, Osteomyelitis acute, Otitis externa, Otitis media, Otitis media acute, Otitis media chronic, Ototoxicity, Overlap syndrome, Parophthalmia, Parotitis, Periarthritis, Pericarditis, Pericarditis adhesive, Pericarditis constrictive, Pericoronitis, Periodontitis, Periostitis, Peritonitis, Pharyngotonsillitis, Plantar fasciitis, Plasma cell balanitis, Pleuropericarditis, Polyarteritis nodosa, Polyglandular autoimmune syndrome type II, Polyserositis, Prostatitis, Pulmonary vasculitis, Purpura non-thrombocytopenic, Raynaud's phenomenon, Renal arteritis, Renal vasculitis, Retinal vasculitis, Retinitis, Retroperitoneal fibrosis, Rheumatoid vasculitis, Rhinitis, Rhinitis allergic, Salivary duct inflammation, Salpingitis, Scleritis, Segmented hyalinising vasculitis, Serositis, Sialadenosis, Sialoadenitis, Sinusitis, Sjogren's syndrome, Sjogren's syndrome, Splenitis, Stomatitis, Stomatitis haemorrhagic, Stomatitis necrotising, Superior limbic keratoconjunctivitis, Susac's syndrome, Systemic immune activation, Tendonitis, Tenosynovitis, Tenosynovitis stenosans, Tonsillitis, Tracheitis, Ulcerative keratitis, Ureteritis, Urethritis, Urethritis noninfective, Urticarial vasculitis, Vascular purpura, Vasculitic rash, Vasculitic rash, Vasculitis, Vasculitis gastrointestinal, Vasculitis necrotising, Venoocclusive liver disease, Vestibular neuronitis, Vogt-Koyanagi-Harada disease, Vulvitis, Vulvovaginitis, Xerophthalmia |
| pancreato-hepatic | pancreatitis | Autoimmune pancreatitis, Immune-mediated pancreatitis, Oedematous pancreatitis, Pancreatic injury, Pancreatic toxicity, Pancreatitis, Pancreatitis acute, Pancreatitis chronic, Pancreatitis haemorrhagic, Pancreatitis necrotising |
| nervous | peripheral  neuropathy | Acute motor axonal neuropathy, Acute motor-sensory axonal neuropathy, Acute polyneuropathy, Autoimmune neuropathy, Axonal and demyelinating polyneuropathy, Axonal neuropathy, Chronic inflammatory demyelinating polyradiculoneuropathy, Demyelinating polyneuropathy, Demyelination, Guillain-Barre syndrome, Immune-mediated neuropathy, Meningoradiculitis, Mononeuritis, Mononeuropathy multiplex, Multifocal motor neuropathy, Neuritis, Neuritis cranial, Optic nerve cupping, Optic neuritis, Optic perineuritis, Papillitis, Polyneuropathy, Polyneuropathy in malignant disease, Sensory ganglionitis, Subacute inflammatory demyelinating polyneuropathy, Toxic neuropathy |
| respiratory | pneumonitis | Acute interstitial pneumonitis, Alveolar lung disease, Alveolitis, Autoimmune lung disease, Bronchiolitis, Bronchitis, Diffuse panbronchiolitis, Eosinophilic bronchitis, Eosinophilic pneumonia, Eosinophilic pneumonia acute, Eosinophilic pneumonia chronic, Granulomatous pneumonitis, Hypersensitivity pneumonitis, Idiopathic interstitial pneumonia, Idiopathic pulmonary fibrosis, Immune-mediated lung disease, Interstitial lung abnormality, Interstitial lung disease, Noninfective bronchitis, Obliterative bronchiolitis, Pneumonitis, Pneumonitis aspiration, Pulmonary alveolar haemorrhage, Pulmonary eosinophilia, Pulmonary fibrosis, Pulmonary toxicity, Sinobronchitis, Tracheobronchitis |
| rheumatology | sarcoidosis | Cardiac sarcoidosis, Cutaneous sarcoidosis, Liver sarcoidosis, Neurosarcoidosis, Ocular sarcoidosis, Pulmonary sarcoidosis, Sarcoid-like reaction, Sarcoidosis, Sarcoidosis of lymph node |
| skin | SCAR | Acute generalised exanthematous pustulosis, Dermatitis bullous, Drug reaction with eosinophilia and systemic symptoms, Epidermal necrosis, Epidermolysis, Nikolsky's sign, Severe cutaneous adverse reaction, SJS-TEN overlap, Stevens-Johnson syndrome, Toxic epidermal necrolysis |
| skin | **Auto-immune** skin bullous | Acquired epidermolysis bullosa, Autoimmune blistering disease, Dermatitis herpetiformis, Epidermolysis bullosa, Lichen planus pemphigoides, Linear IgA disease, Mucous membrane pemphigoid, Pemphigoid, Pemphigus |
| hematology | thrombopenia | Acquired amegakaryocytic thrombocytopenia, Autoimmune pancytopenia, Immune thrombocytopenia |
| endocrine | thyroiditis | Atrophic thyroiditis, Autoimmune hypothyroidism, Autoimmune thyroid disorder, Autoimmune thyroiditis, Basedow's disease, Hashimoto's encephalopathy, Hashitoxicosis, Hyperthyroidism, Hypothyroidism, Immune-mediated hyperthyroidism, Immune-mediated hypothyroidism, Immune-mediated thyroiditis, Myxoedema, Myxoedema coma, Primary hyperthyroidism, Primary hypothyroidism, Silent thyroiditis, Thyroid gland injury, Thyroiditis, Thyroiditis acute, Thyroiditis chronic, Thyroiditis subacute, Thyrotoxic crisis |
| eye | uveitis | Autoimmune uveitis, Immune-mediated uveitis, Iridocyclitis, Iritis, Keratouveitis, Uveitis, Vitritis |
| skin | various skin | Acarodermatitis, Acrodermatitis enteropathica, Acute febrile neutrophilic dermatosis, Alopecia, Alopecia areata, Alopecia totalis, Alopecia universalis, Anal eczema, Angiodermatitis, Annular elastolytic giant cell granuloma, Autoimmune dermatitis, Breast cellulitis, Butterfly rash, Cellulitis, Cellulitis orbital, Chronic actinic dermatitis, Chronic cutaneous lupus erythematosus, Cutaneous lupus erythematosus, Cutaneous vasculitis, Dermatitis, Dermatitis acneiform, Dermatitis allergic, Dermatitis atopic, Dermatitis contact, Dermatitis diaper, Dermatitis exfoliative, Dermatitis exfoliative generalised, Dermatitis psoriasiform, Dermo-hypodermitis, Diffuse alopecia, Drug eruption, Eczema, Eczema nummular, Eczema weeping, Eosinophilic cellulitis, Eosinophilic pustular folliculitis, Erythrodermic psoriasis, Eyelid rash, Folliculitis, Genital rash, Granulomatous dermatitis, Guttate psoriasis, Hand dermatitis ,Immune-mediated dermatitis, Interstitial granulomatous dermatitis, Koebner phenomenon, Lichen nitidus, Lichen planopilaris, Lichen planus, Lichen sclerosus, Lichenification, Morphoea, Nail psoriasis, Nail toxicity, Necrobiosis lipoidica diabeticorum, Necrotising fasciitis, Neurodermatitis, Neutrophilic panniculitis, Nodular rash, Onychomadesis, Oral lichen planus, Oral lichenoid reaction, Palmar erythema, Palmoplantar pustulosis, Panniculitis, Panniculitis lobular, Paraneoplastic dermatosis, Parapsoriasis, Penile dermatitis, Perineal rash, Perioral dermatitis, Periorbital cellulitis, Photodermatosis, Photosensitivity reaction, Pityriasis rubra pilaris, Plantar erythema, Pseudocellulitis, Psoriasis, Pustular psoriasis, Pyoderma gangrenosum, Rash, Rash erythematous, Rash follicular, Rash macular, Rash maculo-papular, Rash morbilliform, Rash papular, Rash papulosquamous, Rash pruritic, Rash pustular, Rash scarlatiniform, Rash vesicular, Reactive perforating collagenosis, Rebound psoriasis, Recall phenomenon, Scleroderma, Scleroderma-like reaction, Scrotal dermatitis, Serum sickness, Skin toxicity, Solar dermatitis, Stasis dermatitis, Subacute cutaneous lupus erythematosus, Subcorneal pustular dermatosis, Superficial inflammatory dermatosis, Symmetrical drug-related intertriginous and flexural exanthema, Systemic lupus erythematosus rash, Systemic scleroderma, Toxic skin eruption, Urticaria chronic, Vulvovaginal rash |
| skin | vitiligo | Achromotrichia acquired, Leukoderma, Pigmentation disorder, Post inflammatory pigmentation change, Skin depigmentation, Skin discolouration, Skin hypopigmentation, Vitiligo |

## Table S2. The Strengthening the Reporting of Observational Studies in Epidemiology (STROBE) checklist: guidelines for reporting observational studies.

## Table S3. Over-reported tandems

All the numbers were calculated within the ICSRs with exposure to ICI (N= 169,753 ICSRs).

Comedications that were co-reported with another suspect comedication were considered “main” when they had the highest ROR value of all comedications for the irAE. Tandems for which the percentage of ICSRs was used as main was <50% were discarded. Rifampicin was mainly (88.9%) reported with isoniazid for hepatitis and its ROR for hepatitis was 9.71 (<12.63 for isoniazid).

Tandems with a comedications used as a treatment of the irAE (such as pyridostigmine for myasthenia) were discarded (percentage cut-off = 1%).

Comedications with the exact same N with ICI of ATC level 4 and ATC level 5, were analyzed only for level 5. The “Percentage used as irAE treatment” is the percentage of ICSRs where the drug indication was a symptom of an irAE (defined as “broad irAE in [PMID:38560659]).

Associations highlighted in grey indicate the 33 relevant selected interactions.

Abbreviations: comed: concomitant medication; NSAIDs: Non Steroidal Anti-Inflammatory Drugs; wo: without ;

| **irAE** | **comedication** | **ROR** | **N_obs_ with comed and irAE** | **N_comedication_** | **N_irAE_** | **Percentage where comed is main** | **Percentage used as irAE treatment** | **ATC level 4 with same N than level 5** |
| --- | --- | --- | --- | --- | --- | --- | --- | --- |
| **diabetes** | sodium-glucose co-transporter 2 (sglt2) inhibitors | 35.77 (3.05-419.87) | 5 | 15 | 1638 | NA | 23.27 | No |
| **enterocolitis** | lansoprazole | 3.78 (1.14-12.54) | 18 | 82 | 8219 | NA | 7.46 | No |
| **hepatitis** | **paracetamol** | **2.76 (1.06-7.20)** | **25** | **211** | **5520** | **92.00** | **0.08** | **No** |
|  | **isoniazid** | **12.63 (3.03-52.69)** | **16** | **42** | **5520** | **100.00** | **NA** | **No** |
|  | carbimazole | 10.25 (1.64-64.19) | 9 | 27 | 5520 | NA | 5.26 | No |
|  | rifampicin | 9.71 (1.58-59.83) | 9 | 28 | 5520 | 11.11 | NA | No |
|  | **ketoconazole** | **20.49 (1.53-274.17)** | **6** | **12** | **5520** | **100.00** | **NA** | **No** |
|  | anilide analgesics and antipyretics | 2.76 (1.06-7.20) | 25 | 211 | 5520 | NA | NA | Yes |
|  | hydrazides for tuberculosis treatment | 12.63 (3.03-52.69) | 16 | 42 | 5520 | NA | NA | Yes |
|  | sulfur-containing imidazole derivatives, antithyroid preparations | 6.83 (1.53-30.58) | 12 | 48 | 5520 | NA | 4.09 | No |
|  | antibiotics, antitubercular | 9.22 (1.52-56.01) | 9 | 29 | 5520 | NA | NA | Yes |
|  | anticorticosteroids | 13.66 (1.28-145.82) | 6 | 15 | 5520 | NA | NA | Yes |
|  | **non-selective monoamine reuptake inhibitors** | **9.56 (1.22-74.79)** | **7** | **22** | **5520** | **100.00** | **0.37** | **No** |
| **myasthenia gravis-like** | pyridostigmine | 177.76 (10.61-2977.5) | 7 | 11 | 1162 | NA | 20.45 | No |
|  | anticholinesterase parasympathomimetics | 203.33 (12.95-3192.58) | 8 | 12 | 1162 | NA | 19.00 | No |
|  | immunoglobulins, normal human | 50.70 (4.32-595.63) | 5 | 15 | 1162 | NA | 12.50 | No |
|  | other nervous system drugs in atc | 24.09 (4.53-128.03) | 9 | 47 | 1162 | NA | 18.75 | No |
| **myocarditis** | influenza vaccine* | 26.53 (3.92-179.38) | 8 | 26 | 1960 | NA | 0.00 | YES |
|  | **influenza vaccines*** | **22.74 (3.51-147.37)** | **8** | **29** | **1960** | **100.00** | **0.00** | **No** |
| **myositis** | **atorvastatin** | **10.93 (2.96-40.32)** | **14** | **94** | **1880** | **57.14** | **0.32** | **No** |
|  | **rosuvastatin** | **16.76 (2.47-113.79)** | **7** | **33** | **1880** | **100.00** | **0.35** | **No** |
|  | **simvastatin** | **13.33 (1.76-100.83)** | **6** | **34** | **1880** | **100.00** | **0.34** | **No** |
|  | **statins** | **9.41 (3.50-25.30)** | **24** | **184** | **1880** | **100.00** | **0.30** | **No** |
| **nephritis** | **omeprazole** | **55.71 (28.71-108.11)** | **89** | **189** | **1949** | **100.00** | **0.07** | **No** |
|  | **pantoprazole** | **35.82 (13.30-96.52)** | **33** | **89** | **1949** | **100.00** | **0.38** | **No** |
|  | **ibuprofen** | **27.82 (8.93-86.67)** | **23** | **73** | **1949** | **78.26** | **0.00** | **No** |
|  | ciprofloxacin | 24.49 (4.90-122.52) | 11 | 38 | 1949 | NA | 1.18 | No |
|  | **lansoprazole** | **6.49 (1.22-34.62)** | **8** | **82** | **1949** | **100.00** | **0.00** | **No** |
|  | **esomeprazole** | **7.50 (1.23-45.52)** | **7** | **63** | **1949** | **100.00** | **0.00** | **No** |
|  | furosemide | 6.46 (1.08-38.67) | 7 | 72 | 1949 | NA | 1.95 | No |
|  | losartan | 10.50 (1.66-66.31) | 7 | 47 | 1949 | 0.00 | 0.25 | No |
|  | **rabeprazole** | **52.50 (5.12-537.97)** | **7** | **15** | **1949** | **100.00** | **0.00** | **No** |
|  | celecoxib | 14.99 (1.92-116.77) | 6 | 30 | 1949 | 16.67 | 0.00 | No |
|  | pravastatin | 17.63 (1.79-173.64) | 5 | 22 | 1949 | NA | 0.52 | No |
|  | **proton pump inhibitors** | **29.62 (18.61-47.14)** | **144** | **457** | **1949** | **100.00** | **0.16** | **No** |
|  | **NSAIDs, propionic acid derivatives** | **10.47 (4.15-26.41)** | **28** | **190** | **1949** | **71.43** | **0.00** | **No** |
|  | fluoroquinolone antibacterials, systemic | 6.56 (1.67-25.79) | 12 | 122 | 1949 | NA | 0.59 | No |
|  | Angiotensin 2 receptor blockers | 7.65 (2.22-26.32) | 15 | 133 | 1949 | 13.33 | 0.00 | No |
|  | coxibs | 13.33 (1.75-101.44) | 6 | 33 | 1949 | NA | NA | Yes |
| **pneumonitis** | **amiodarone** | **10.01 (3.75-26.71)** | **41** | **84** | **10339** | **100.00** | **0.00** | **No** |
|  | sulfamethoxazole | 2.37 (1.02-5.49) | 35 | 190 | 10339 | NA | 13.27 | No |
|  | trimethoprim | 2.34 (1.01-5.42) | 35 | 192 | 10339 | NA | 12.88 | No |
|  | antiarrhythmics, class III | 9.78 (3.69-25.96) | 41 | 85 | 10339 | NA | NA | Yes |
|  | intermediate-acting sulfonamides | 2.37 (1.02-5.49) | 35 | 190 | 10339 | NA | NA | Yes |
|  | trimethoprim and derivatives | 2.34 (1.01-5.42) | 35 | 192 | 10339 | NA | NA | Yes |
| **SCAR** | **sulfamethoxazole** | **31.31 (13.32-73.61)** | **35** | **190** | **882** | **91.43** | **0.00** | **No** |
|  | trimethoprim | 30.91 (13.16-72.60) | 35 | 192 | 882 | 0.00 | 0.00 | No |
|  | amoxicillin | 13.73 (4.03-46.82) | 15 | 163 | 882 | NA | 0.51 | No |
|  | **clavulanic acid** | **18.12 (4.77-68.89)** | **13** | **110** | **882** | **92.31** | **0.47** | **No** |
|  | **allopurinol** | **57.11 (11.27-289.39)** | **11** | **37** | **882** | **100.00** | **0.00** | **No** |
|  | piperacillin | 32.88 (6.70-161.49) | 10 | 51 | 882 | NA | 5.52 | No |
|  | tazobactam | 31.90 (5.99-169.79) | 9 | 47 | 882 | NA | 5.80 | No |
|  | lansoprazole | 14.54 (2.71-77.89) | 8 | 82 | 882 | 37.50 | 0.00 | No |
|  | **paracetamol** | **5.29 (1.04-26.92)** | **8** | **211** | **882** | **50.00** | **0.02** | **No** |
|  | **loxoprofen** | **17.41 (2.85-106.50)** | **7** | **61** | **882** | **71.43** | **0.00** | **No** |
|  | **pantoprazole** | **11.47 (1.94-67.66)** | **7** | **89** | **882** | **85.71** | **0.00** | **No** |
|  | esomeprazole | 14.12 (2.04-97.68) | 6 | 63 | 882 | 16.67 | 0.00 | No |
|  | **levetiracetam** | **14.91 (2.14-103.64)** | **6** | **60** | **882** | **100.00** | **0.00** | **No** |
|  | ibuprofen | 9.85 (1.22-79.42) | 5 | 73 | 882 | 40.00 | 0.00 | No |
|  | levofloxacin | 13.40 (1.62-110.81) | 5 | 55 | 882 | NA | 0.60 | No |
|  | **ondansetron** | **10.99 (1.35-89.26)** | **5** | **66** | **882** | **80.00** | **0.00** | **No** |
|  | antiinfectives for systemic use | 8.13 (4.85-13.64) | 88 | 1669 | 882 | NA | NA | Yes |
|  | intermediate-acting sulfonamides | 31.31 (13.32-73.61) | 35 | 190 | 882 | NA | NA | Yes |
|  | trimethoprim and derivatives, systemic antibacterials | 30.91 (13.16-72.60) | 35 | 192 | 882 | NA | NA | Yes |
|  | penicillins with extended spectrum | 16.46 (6.28-43.14) | 25 | 233 | 882 | NA | 2.09 | No |
|  | combinations of penicillins, incl. beta-lactamase inhibitors | 16.74 (4.43-63.26) | 13 | 118 | 882 | NA | NA | Yes |
|  | preparations inhibiting uric acid production | 51.20 (10.36-253.06) | 11 | 40 | 882 | NA | NA | Yes |
|  | beta-lactamase inhibitors, systemic penicillins | 25.25 (4.90-129.99) | 9 | 57 | 882 | NA | NA | No |
|  | **proton pump inhibitors** | **8.61 (3.48-21.26)** | **27** | **457** | **882** | **59.26** | **0.00** | **No** |
|  | anilide analgesics and antipyretics | 5.29 (1.04-26.92) | 8 | 211 | 882 | NA | NA | No |
|  | **NSAIDs, propionic acid derivatives** | **9.10 (2.36-35.04)** | **12** | **190** | **882** | **58.33** | **0.00** | **No** |
|  | **Other antiepileptics** | **5.69 (1.12-28.98)** | **8** | **197** | **882** | **87.50** | **0.00** | **No** |
|  | fluoroquinolone antibacterial, systemic | 16.13 (4.28-60.78) | 13 | 122 | 882 | NA | 0.59 | No |
|  | **serotonin (5ht3) antagonists** | **10.81 (1.84-63.52)** | **7** | **94** | **882** | **57.14** | **NA** | **No** |
|  | antibiotics, intestinal | 15.96 (1.90-134.34) | 5 | 47 | 882 | NA | 4.51 | No |
| **skin bullous** | **sitagliptin** | **28.92 (3.89-214.74)** | **6** | **38** | **769** | **100.00** | **0.00** | **No** |
|  | **vildagliptin** | **256.79 (9.62-6853.76)** | **5** | **8** | **769** | **100.00** | **0.00** | **No** |
|  | **dipeptidyl peptidase 4 inhibitors** | **46.42 (11.71-184.05)** | **14** | **61** | **769** | **100.00** | **0.00** | **No** |
| thyroiditis | thiamazole | 13.76 (1.93-97.97) | 10 | 21 | 7354 | NA | 65.26 | No |
|  | thyroid hormones | 3.98 (1.29-12.34) | 20 | 96 | 7354 | NA | 30.78 | No |
|  | sulfur-containing imidazole derivatives, antithyroid preparations | 8.30 (2.14-32.25) | 17 | 48 | 7354 | NA | 57.89 | No |
| various skin | amoxicillin | 5.56 (2.68-11.54) | 60 | 163 | 11289 | NA | 26.16 | No |
|  | clavulanic acid | 4.83 (1.95-11.96) | 37 | 110 | 11289 | NA | 28.77 | No |
|  | sulfamethoxazole | 2.30 (1.01-5.24) | 37 | 190 | 11289 | NA | NA | No |
|  | penicillins with extended spectrum | 4.10 (2.15-7.80) | 70 | 233 | 11289 | NA | NA | Yes |
|  | combinations of penicillins, incl. beta-lactamase inhibitors | 4.70 (1.95-11.33) | 39 | 118 | 11289 | NA | 27.32 | No |
|  | intermediate-acting sulfonamides | 2.30 (1.01-5.24) | 37 | 190 | 11289 | NA | 25.65 | No |

* ”Influenza vaccines” with an “s” refers to the ATC level 4 group of influenza vaccines (ATC J07BB), and without an “s” for the ATC level 5 “influenza vaccine” not othewerwise specified

## Table S4. Calculation of the reporting odds ratio (ROR) ratio (ROR_ratio_).

To test a potential synergy between the comedications and the presence of ICIs in the occurrence of irAEs, we calculated the ROR within the ICI database and we compared it to the ROR of the whole VigiBase with the exclusion of ICSRs with an exposure to an ICI (ROR_VigiBase without ICI_=ROR_VBwoICI_). The ROR_ratio_ was the ratio of ROR_ICI_ and ROR_woICI_.

$${ROR}_{ratio}=\frac{{ROR}_{ICI}}{{ROR}_{VBwoICI}}$$

The number of ICSRs in the whole database without ICI was N=36,401,565.

N_obs_ is the number observed with comedication and irAE within the population. N_comed_ is the number of ICSRs with the comedication in the specified population declared as “suspect or interacting”. N_irAE_ is the number of ICSRs with the immune-related adverse event in the ICI population. N_(ir)AE_ is the number of adverse events that was found in the whole database using the “preferred term” used to define the irAE. Since it is mainly not used in the context of immunotherapy in the full VigiBase, the “immune-related” terminology is improper.

Abbreviations: wo: without ; NSAIDs: Non Steroidal Anti-Inflammatory Drugs;

|  |  | **ICSRs with an ICI**  **(N=169,753)** | | | |  | **ICSRs in VigiBase without an ICI (N=36,231,812)** | | | |  |  |
| --- | --- | --- | --- | --- | --- | --- | --- | --- | --- | --- | --- | --- |
| **irAE** | **comedication** | **N_obs_** | **N_comed_** | **N_irAE_** | **ROR_ICI_** |  | **N_obs_** | **N_comed_** | **N_(ir)AE_** | **ROR_VBwoICI_** |  | **ROR_ratio_** |
| **hepatitis** | ketoconazole | 6 | 12 | 5520 | 20.49 [1.53-274.17] |  | 628 | 9457 | 155338 | 16.61 (15.31 - 18.01) |  | 1.23 (0.15-10) |
|  | isoniazid | 16 | 42 | 5520 | 12.63 [3.03-52.69] |  | 6784 | 60422 | 155338 | 30.71 (29.93 - 31.51) |  | 0.41 (0.04-4.2) |
|  | paracetamol | 25 | 211 | 5520 | 2.76 [1.06-7.20] |  | 6818 | 203412 | 155338 | 8.39 (8.19 - 8.60) |  | 0.33 (0.07-1.6) |
|  | non-selective monoamine reuptake inhibitors | 7 | 22 | 5520 | 9.56 [1.22-74.79] |  | 1513 | 89623 | 155338 | 4.02 (3.82 - 4.23) |  | 2.38 (0.084-67) |
| **myocarditis** | **influenza vaccines** | **8** | **29** | **1960** | **22.74 [3.51-147.37]** |  | **318** | **375609** | **46682** | **0.66 (0.59 - 0.73)** |  | **34.45 (1.7-720)** |
| **myositis** | statins | 14 | 94 | 1880 | 9.41 [3.50-25.30] |  | 26316 | 361012 | 64130 | 74.61 (73.42 - 75.82) |  | 0.13 (0.025-0.63) |
|  | rosuvastatin | 7 | 33 | 1880 | 16.76 [2.47-113.79] |  | 3393 | 70918 | 64130 | 29.91 (28.87 - 30.99) |  | 0.56 (0.025-13) |
|  | simvastatin | 6 | 34 | 1880 | 13.33 [1.76-100.83] |  | 7553 | 78941 | 64130 | 67.60 (65.92 - 69.32) |  | 0.05 (0.0018-1.3) |
|  | atorvastatin | 14 | 94 | 1880 | 10.93 [3.00-40.32] |  | 8220 | 150820 | 64130 | 37.20 (36.32 - 38.09) |  | 0.29 (0.035-2.4) |
| **nephritis** | omeprazole | 89 | 189 | 1949 | 55.71 [28.71-108.11] |  | 4529 | 136078 | 48574 | 28.22 (27.36 - 29.11) |  | 1.97 (0.67-5.8) |
|  | rabeprazole | 7 | 15 | 1949 | 52.50 [5.12-537.97] |  | 1105 | 19255 | 48574 | 46.45 (43.68 - 49.39) |  | 1.13 (0.026-49) |
|  | pantoprazole | 33 | 89 | 1949 | 35.82 [13.30-96.52] |  | 3361 | 78853 | 48574 | 35.61 (34.35 - 36.90) |  | 1.01 (0.2-5) |
|  | **ibuprofen** | **23** | **73** | **1949** | **27.82 [8.93-86.67]** |  | **909** | **193966** | **48574** | **3.56 (3.33 - 3.80)** |  | **7.81 (1.2-50)** |
|  | esomeprazole | 7 | 63 | 1949 | 7.50 [1.23-45.52] |  | 3750 | 110000 | 48574 | 28.45 (27.50 - 29.43) |  | 0.26 (0.014-4.9) |
|  | lansoprazole | 8 | 82 | 1949 | 6.49 [1.22-34.62] |  | 3815 | 65440 | 48574 | 50.03 (48.36 - 51.76) |  | 0.13 (0.0086-2) |
|  | proton pump inhibitors | 144 | 457 | 1949 | 29.62 [18.61-47.14] |  | 6135 | 309091 | 48574 | 17.15 (16.69 - 17.61) |  | 1.73 (0.81-3.7) |
|  | NSAIDs, propionic acid derivatives | 28 | 190 | 1949 | 10.47 [4.15-26.41] |  | 2097 | 448457 | 48574 | 3.62 (3.46 - 3.78) |  | 2.89 (0.64-13) |
| **pneumonitis** | amiodarone | 41 | 84 | 10339 | 10.01 [3.75-26.71] |  | 5506 | 50843 | 155839 | 29.15 (28.33 - 29.99) |  | 0.34 (0.07-1.7) |
| **SCAR** | allopurinol | 11 | 37 | 882 | 57.11 [11.27-289.39] |  | 7901 | 42871 | 125882 | 69.17 (67.46 - 70.93) |  | 0.83 (0.059-11) |
|  | sulfamethoxazole | 35 | 190 | 882 | 31.31 [13.32-73.61] |  | 8430 | 138743 | 125882 | 19.84 (19.40 - 20.30) |  | 1.58 (0.39-6.3) |
|  | clavulanic acid | 13 | 110 | 882 | 18.12 [4.77-68.89] |  | 3137 | 185056 | 125882 | 5.05 (4.88 - 5.24) |  | 3.59 (0.41-31) |
|  | loxoprofen | 7 | 61 | 882 | 17.41 [2.85-106.50] |  | 366 | 23163 | 125882 | 4.62 (4.17 - 5.13) |  | 3.77 (0.2-71) |
|  | levetiracetam | 6 | 60 | 882 | 14.91 [2.15-103.64] |  | 955 | 53464 | 125882 | 5.26 (4.93 - 5.60) |  | 2.84 (0.12-66) |
|  | pantoprazole | 7 | 89 | 882 | 11.5 [1.9-67.7] |  | 616 | 78853 | 125882 | 2.27 (2.09 - 2.46) |  | 5.06 (0.28-90) |
|  | ondansetron | 5 | 66 | 882 | 11.00 [1.40-89.30] |  | 150 | 26654 | 125882 | 1.63 (1.39 - 1.91) |  | 6.76 (0.22-200) |
|  | paracetamol | 25 | 211 | 882 | 5.3 [1-26.9] |  | 3206 | 203412 | 125882 | 4.69 (4.53 - 4.86) |  | 1.13 (0.081-16) |
|  | serotonin (5HT3) antagonist | 7 | 94 | 882 | 10.81 [1.84-63.52] |  | 184 | 53163 | 125882 | 1.00 (0.86 - 1.15) |  | 10.81 (0.61-190) |
|  | NSAIDs, propionic acid derivatives | 28 | 190 | 882 | 10.47 [4.15-26.41] |  | 4608 | 448457 | 125882 | 3.06 (2.97 - 3.15) |  | 3.42 (0.33-27) |
|  | proton pump inhibitors | 27 | 457 | 882 | 8.61 [3.48-21.26] |  | 2503 | 309091 | 125882 | 2.37 (2.28 - 2.47) |  | 3.63 (0.84-16) |
|  | other antiepileptic | 8 | 197 | 882 | 5.69 [1.12-28.98] |  | 7444 | 513289 | 125882 | 4.43 (4.33 - 4.54) |  | 1.28 (0.075-66) |
| **skin bullous** | vildagliptin | 5 | 8 | 769 | 256.79 [9.62-6853.76] |  | 768 | 10362 | 9900 | 317.88 (294.53 - 343.09) |  | 0.81 (0.0039-170) |
|  | sitagliptin | 6 | 38 | 769 | 28.92 [3.89-214.74] |  | 401 | 34274 | 9900 | 45.16 (40.85 - 49.94) |  | 0.64 (0.025-17) |
|  | dipeptidyl peptidase 4 inhibitors | 14 | 61 | 769 | 46.42 [11.71-184.05] |  | 1993 | 66090 | 9900 | 142.39 (135.48 - 149.65) |  | 0.33 (0.035-3.1) |

## Table S5. Multivariate analysis performed on the relevant associations identified.

A multivariate logistic regression analysis was performed on the 33 identified tandems with a suspected interaction risk. The adjustment factors for this regression were the factors significantly associated with the irAE in the stepwise analysis [PMID:38560659], with the addition of the tested comedication.

| irAE | comedication | OR multivariate |
| --- | --- | --- |
| hepatitis | isoniazid | 34.45 (15.71-77.46) |
|  | paracetamol | 4.69 (2.95-7.16) |
|  | ketoconazole | 54.62 (15.97-194.01) |
|  | non-selective monoamine reuptake inhibitors | 12.22 (4.55-29.94) |
| myocarditis | influenza vaccines | 31.44 (11.91-76.93) |
| myositis | statins | 20.24 (13.07-30.44) |
|  | rosuvastatin | 31.49 (11.69-75.75) |
|  | atorvastatin | 19.40 (10.16-34.73) |
|  | simvastatin | 17.21 (6.13-41.62) |
| nephritis | omeprazole | 131.64 (94.34-184.58) |
|  | rabeprazole | 95.25 (32.33-274.18) |
|  | pantoprazole | 51.41 (31.94-82.03) |
|  | esomeprazole | 10.46 (4.26-22.03) |
|  | lansoprazole | 9.62 (4.21-19.16) |
|  | ibuprofen | 46.63 (26.84-79.51) |
|  | proton pump inhibitors | 51.75 (41.53-64.27) |
|  | nsaids, propionic acid derivatives | 20.80 (13.39-31.23) |
| pneumonitis | amiodarone | 14.63 (9.27-23.10) |
| SCAR | proton pump inhibitors | 12.03 (7.88-17.59) |
|  | pantoprazole | 18.72 (8.63-35.96) |
|  | serotonin (5-ht3) agonists | 19.15 (8.75-37.24) |
|  | ondansetron | 10.48 (3.62-24.02) |
|  | clavulanic acid | 24.80 (12.96-43.93) |
|  | sulfamethoxazole | 44.76 (30.11-64.96) |
|  | loxoprofen | 25.27 (10.29-53.35) |
|  | allopurinol | 100.79 (45.35-213.39) |
|  | paracetamol | 7.48 (3.53-13.85) |
|  | levetiracetam | 19.04 (7.20-41.93) |
|  | nsaids, propionic acid derivatives | 15.79 (8.65-26.57) |
|  | other antiepileptics | 2.01 (0.33-6.35) |
| skin bullous | dipeptidyl peptidase 4 inhibitors | 59.65 (30.04-111.89) |
|  | vildagliptin | 409.41 (77.04-3102.54) |
|  | sitagliptin | 34.61 (12.58-80.78) |
